# Supplementary material for: Anthropogenic influence on extreme precipitation over global land areas seen in multiple observational datasets
Source: Nat Commun. 2021 Jul 6;12:3944. doi: 10.1038/s41467-021-24262-x (PMC8260601; doi:10.1038/s41467-021-24262-x)
Supplement: Supplementary file 1 — Supplementary Information [file 41467_2021_24262_MOESM1_ESM.pdf]

Supporting Information for

Anthropogenic influence on extreme precipitation over global land areas seen in multiple observational datasets

Gavin D. Madakumbura[1*], Chad W. Thackeray[1], Jesse Norris[1], Naomi Goldenson[1] and Alex Hall[1]

[1]Department of Atmospheric and Oceanic Sciences, University of California — Los Angeles, Los Angeles, CA, USA.

[*]correspondence e-mail address: gavindayanga@ucla.edu

Contents of this file:

1. Supplementary Text

2. Supplementary Table 1-3

3. Supplementary Figures 1-9

Supplementary Text:

1. More on Layer-wise Relevance Propagation

The $\alpha\beta$-rule rule with $\alpha = 1$ and $\beta = 0$ (LRP$_{\alpha 1\beta 0}$) only considers the information which positively contributes to the final decision. For regression tasks such as the problem at hand here, inputs which contribute to a decrease in $f(\mathbf{x})$ (i.e. an earlier predicted year; negative relevance) are equally as important as inputs which contribute to an increase (i.e. a later predicted year; positive relevance) to understand what the ANN has learned. Moreover, when $\alpha > 1$, the $\alpha\beta$-rule might not conserve the relevance from the output value back to the input layer. For these reasons, ref. 1 pointed out that caution should be exercised when applying the $\alpha\beta$-rule with 1) $\alpha = 1$ for regression and 2) $\alpha > 1$ in general. This is mainly because the interpretation of relevance heatmaps can be more subjective in these cases. We find that for our simple ANN, applying LRP$_{\alpha 2\beta 1}$ results in a 1:1 relationship between the resultant relevance heatmaps and $f(\mathbf{x})$ for each input (Supplementary Fig. 4d). This allows the visualization of input that contributes to a decrease in $f(\mathbf{x})$ while maintaining a direct relationship between the ANN predicted value and LRP heatmaps. Therefore, we proceed with rescaled relevance heatmaps derived from LRP$_{\alpha 2\beta 1}$ for interpreting our ANN. We also found qualitatively similar relevance heatmaps with the basic relevance propagation rule LRPz, which does not treat negative and positive pre-activations separately. More details on LRP can be found in previous work (ref. 1-4). For a toy example of LRP, we refer to ref. 5.

2. Role of model uncertainty in detecting the anthropogenic influence

To assess the influence of model uncertainty in detecting the signal, we redid the analysis, but including a widely used highly quality controlled HadEX3 dataset[6], which along with its predecessors have been used in traditional detection and attribution of extreme precipitation[7,8]. HadEX3 and its

predecessors are considered as a more reliable dataset than the other observational estimates used in this study, but do not provide full global coverage. Therefore, analyses were done for all GCMs and observations, just over the regions with a continuous data coverage in HadEX3 for the period 1979-2018 (Supplementary Fig. 5). Two separate analyses were conducted. The first analysis was similar to the main analysis (Figures 1-4), using multimodel simulations to train the ANN (Supplementary Fig. 6) to include the model uncertainty. The second was done to assess the role of model uncertainty. The ideal ANN input dataset in this case would be a large ensemble of realizations which have a time evolution of the ensemble mean equivalent to the multimodel mean of the CMIP models used in the first analysis. The difference between realizations in this case represents the natural variability, as opposed to the first case in which it includes model uncertainty as well. We found CESM large ensemble simulations[9] suitable for this task. We used 40 initial condition perturbed ensemble members from the dataset for the period 1920-2099. Simulations follow similar forcing as CMIP5 models described in methods. To follow the same ANN training process as the first step, we used 26 ensemble members for training, 9 ensemble members for validation and the rest (5) for testing. Thereafter, the analysis is identical to the main analysis (Supplementary Fig. 7).

When the model uncertainty is included, observations and reanalysis fail to identify the anthropogenic influence for the selected domain (Supplementary Fig. 6), whereas when the model uncertainty is not included signal is detected in 9 out of 12 datasets (Supplementary Fig. 7). This suggests that when the model uncertainty is considered, the power of detecting the anthropogenic influence decreases.

Similar behavior in reanalysis and observations and the difference compared to testing data in these results (Supplementary Fig. 6,7) also justifies the use of reanalysis as an alternative observation in assessing the anthropogenic influence, as argued in previous studies (e.g. ref. 10).

3. Sources of the spread in the signal of observations

For observational datasets used in the main text, the absolute value of the predicted year shows a wide range of values, with an overall underestimation compared to GCMs (Supplementary Fig. 3). A composite difference of the relevance and Rx1day between observations and testing models reveal that different regions contribute to this result (Supplementary Fig. 8). In observations, a lower relevance compared to GCMs can be seen over Asia and North America (Supplementary Fig. 8a,c,e,g). These patterns correspond to an underestimation of Rx1day in the historical observational record compared to GCMs (Supplementary Fig. 8b,d,f,h). Among the observations, the predicted year for MSWEP is the highest, which is due to having higher Rx1day over Greenland, Alaska and East Russia.

To investigate the differences in the anthropogenic signal in the observations and reanalyses, we first calculated the linear trend of Rx1day for each grid cell and weighted that by the normalized relevance for grid cells with a positive relevance (Supplementary Fig. 9). A simple explanation for this difference is that when more pixels with a positive relevance show an increase in Rx1day there is an increase in the predicted year. Confirming this, datasets with a smaller anthropogenic signal (e.g. ERA5, and CFSR, as shown in Figure 4) have a smaller number of grid cells with an increasing relevance-weighted trend in Rx1day compared to the rest of the datasets (Supplementary Fig. 9).

Supplementary Table 1: Observations and reanalysis used in this study. Data for 1982-2015 was used as that is the common period for all datasets considered here. Trend estimates are the global terrestrial area averaged Rx1day linear trend. Trend and the statistical significance were obtained by using the Theil-Sen estimator[11,12] and modified Mann-Kendall trend test[13], respectively.

	dataset	trend estimates		
		trend (at 99%)	slope (mm/day/yr)	p value
1	MSWEP.v2	No trend	0.02	0.14
2	GPCC FDD 2018	No trend	0.02	0.53
3	REGEN_ALL	Increasing	0.07	1.5×10^{-7}
4	REGEN_LONG	Increasing	0.05	4.3×10^{-6}
5	ERA5	Increasing	0.06	5.7×10^{-8}
6	JRA55	Increasing	0.02	1.5×10^{-3}
7	MERRA2	Increasing	0.09	6.5×10^{-14}
8	CFSR	No trend	0.08	0.05
9	W5E5	Increasing	0.09	8.9×10^{-16}
10	NCEP2	No trend	0.02	0.18
11	20CRv3	Increasing	0.05	1.7×10^{-5}
	CMIP5/6	Increasing in 36 out of 44 models	0.01 - 0.07	2.2×10^{-16} - 0.33

Supplementary Table 2: CMIP models and the ensemble variant used in this study.

	CMIP version	Model name	Ensemble variant
1	CMIP6	ACCESS-CM2	r1i1p1f1
2	CMIP6	ACCESS-ESM1-5	r1i1p1f1
3	CMIP6	CNRM-CM6-1-HR	r1i1p1f2
4	CMIP6	CNRM-CM6-1	r1i1p1f2
5	CMIP6	CNRM-ESM2-1	r1i1p1f2
6	CMIP6	CanESM5	r1i1p1f1
7	CMIP6	EC-Earth3-Veg	r1i1p1f1
8	CMIP6	EC-Earth3	r1i1p1f1
9	CMIP6	GFDL-CM4	r1i1p1f1
10	CMIP6	GFDL-ESM4	r1i1p1f1
11	CMIP6	HadGEM3-GC31-LL	r1i1p1f3
12	CMIP6	INM-CM4-8	r1i1p1f1
13	CMIP6	INM-CM5-0	r1i1p1f1
14	CMIP6	IPSL-CM6A-LR	r1i1p1f1
15	CMIP6	MIROC-ES2L	r1i1p1f2
16	CMIP6	MIROC6	r1i1p1f1
17	CMIP6	MPI-ESM1-2-HR	r1i1p1f1
18	CMIP6	MPI-ESM1-2-LR	r1i1p1f1
19	CMIP6	MPI-ESM2-0	r1i1p1f1
20	CMIP6	NorESM2-LM	r1i1p1f1
21	CMIP6	NorESM2-MM	r1i1p1f1
22	CMIP6	UKESM1-0-LL	r1i1p1f2
23	CMIP5	ACCESS1-3	r1i1p1
24	CMIP5	CanESM2	r2i1p1
25	CMIP5	CMCC-CESM	r1i1p1
26	CMIP5	CMCC-CM	r1i1p1
27	CMIP5	CNRM-CM5	r1i1p1
28	CMIP5	CSIRO-Mk3-6-0	r1i1p1
29	CMIP5	EC-EARTH	r1i1p1
30	CMIP5	GFDL-CM3	r1i1p1
31	CMIP5	HadGEM2-AO	r1i1p1
32	CMIP5	HadGEM2-CC	r1i1p1
33	CMIP5	HadGEM2-ES	r1i1p1
34	CMIP5	inmcm4	r1i1p1
35	CMIP5	IPSL-CM5A-LR	r1i1p1
36	CMIP5	IPSL-CM5A-MR	r1i1p1
37	CMIP5	IPSL-CM5B-LR	r1i1p1
38	CMIP5	MIROC5	r1i1p1
39	CMIP5	MIROC-ESM-CHEM	r1i1p1
40	CMIP5	MIROC-ESM	r1i1p1
41	CMIP5	MPI-ESM-LR	r1i1p1
42	CMIP5	MPI-ESM-MR	r1i1p1
43	CMIP5	MRI-CGCM3	r1i1p1
44	CMIP5	NorESM1-M	r1i1p1

Supplementary Table 3: CMIP6 preindustrial control simulations (models and the ensemble variants) used in this study.

	Model name	Ensemble variant
1	ACCESS-CM2	r1i1p1f1
2	ACCESS-ESM1-5	r1i1p1f1
3	CNRM-CM6-1	r1i1p1f2
4	CNRM-ESM2-1	r1i1p1f2
5	CanESM5	r1i1p1f1
6	EC-Earth3-Veg	r1i1p1f1
7	EC-Earth3	r1i1p1f1
8	GFDL-CM4	r1i1p1f1
9	GFDL-ESM4	r1i1p1f1
10	HadGEM3-	r1i1p1f3
11	INM-CM4-8	r1i1p1f1
12	INM-CM5-0	r1i1p1f1
13	IPSL-CM6A-LR	r1i1p1f1
14	MIROC-ES2L	r1i1p1f2
15	MIROC6	r1i1p1f1
16	MPI-ESM1-2-HR	r1i1p1f1
17	MPI-ESM1-2-LR	r1i1p1f1
18	NorESM2-LM	r1i1p1f1
19	NorESM2-MM	r1i1p1f1
20	UKESM1-0-LL	r1i1p1f2

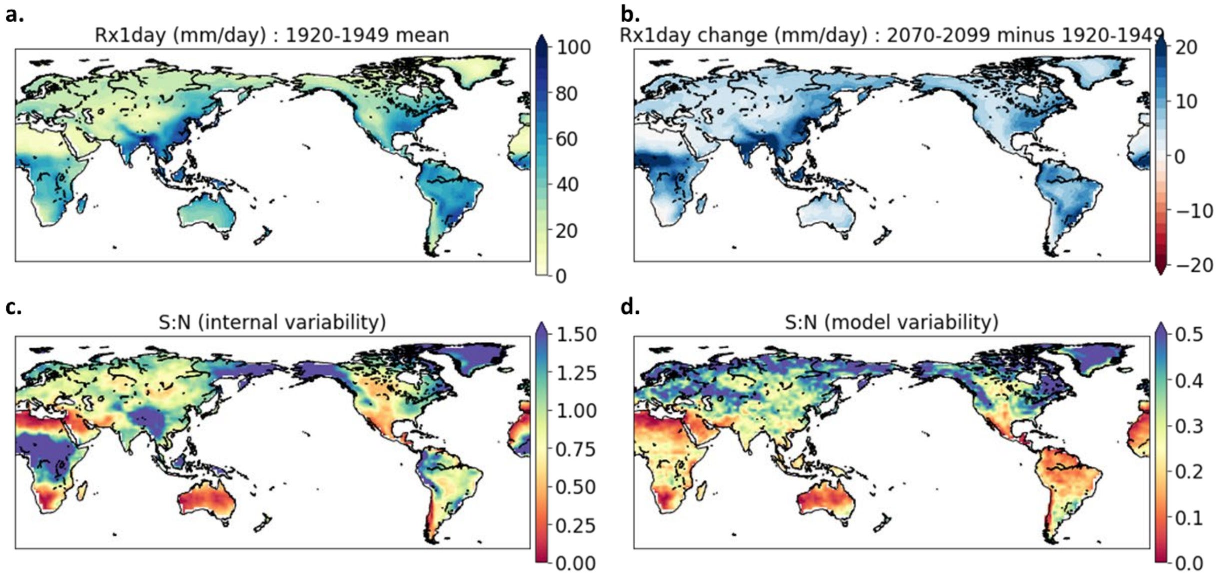

Supplementary Fig. 1. Simulated changes in Rx1day.

(a) Climatology of Rx1day for 1920-1949 calculated as the multimodel mean and the time mean. (b) Multimodel mean difference of Rx1day between 2070-2099 and 1920-1949. (c-d) Signal to noise ratios (S:N) for noise from internal variability (c) and inter-model variability (d). Signal is calculated as the multimodel mean of the Rx1day difference between the periods 2070-2099 and 1920-1949. Noise from the internal variability is calculated as the multimodel mean of the standard deviation during 1920-1949. Noise from the model variability is calculated as the inter-model standard deviation of the signal of each model.

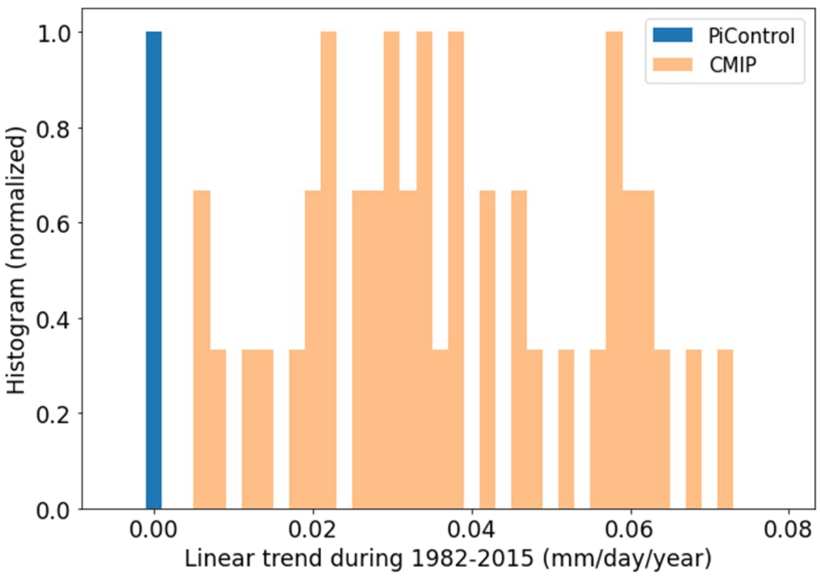

Supplementary Fig. 2. Simulated trends of forced and unforced historical globally averaged Rx1day. Histogram of global averaged Rx1day trend during 1982-2015 in forced historical CMIP simulations (orange). Blue values are from 220 non-overlapping 34-year segments from CMIP6 PiControl simulations to represent the natural variability. Bin values range from -0.005 to 0.08 with a bin width of 0.002 (all values are in mm/day/year). Trend was obtained by using the Theil-Sen estimator[11,12]. The two distributions (orange and blue) are significantly different at $p < 0.001$ (Kolmogorov–Smirnov test).

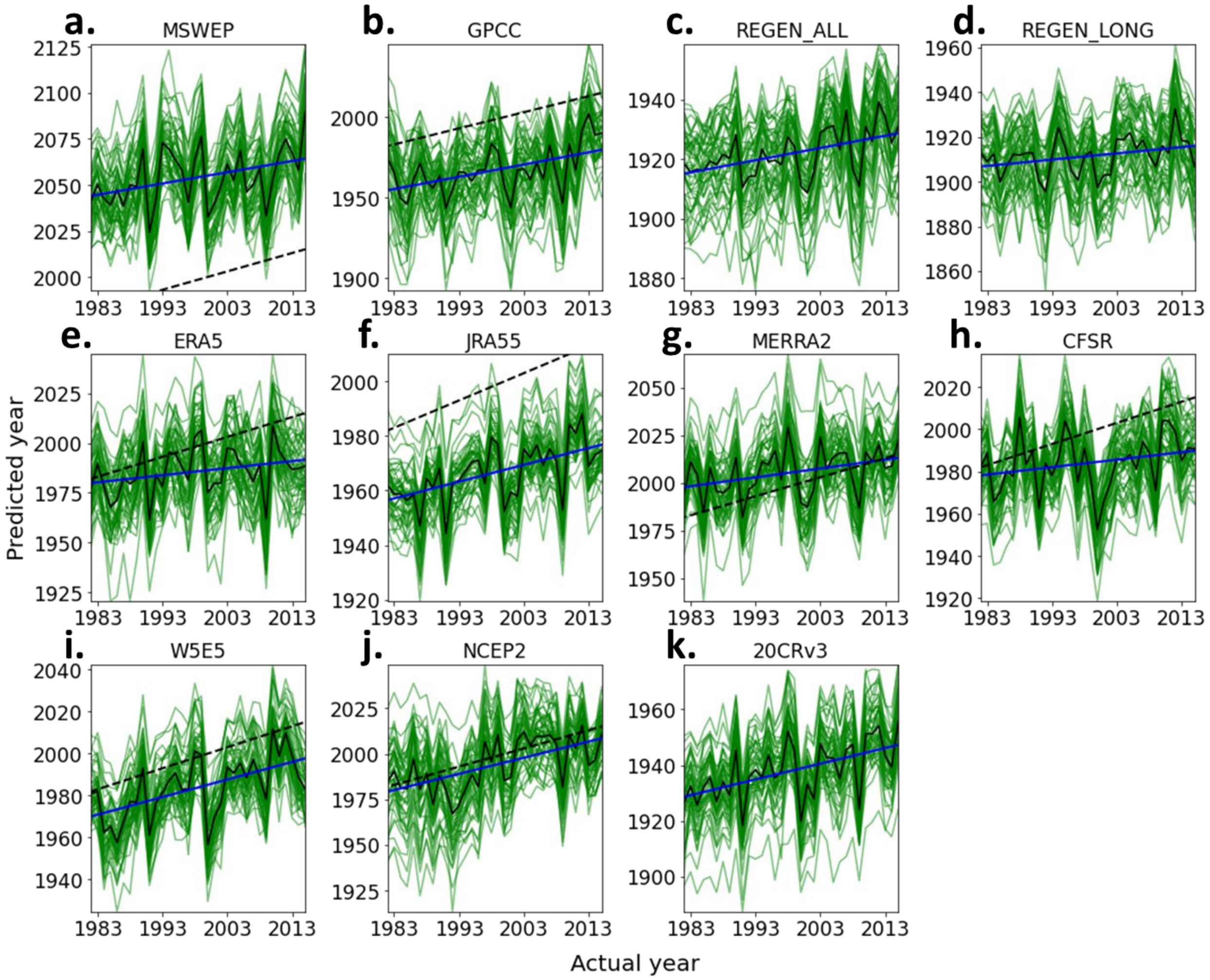

Supplementary Fig. 3. Predicted year for historical Rx1day records by 51 different ANNs. For datasets MSWEP (a), GPCC (b), REGEN_ALL (c), REGEN_LONG (d), ERA5 (e), JRA55 (f), MERRA2 (g), CFSR (h), W5E5 (i), NCEP2 (j),20CRv3 (k). Black dashed line is the 1:1 line. Blue line is the mean slope. Note that the y-axis limits are set as the maximum and minimum values of each figure.

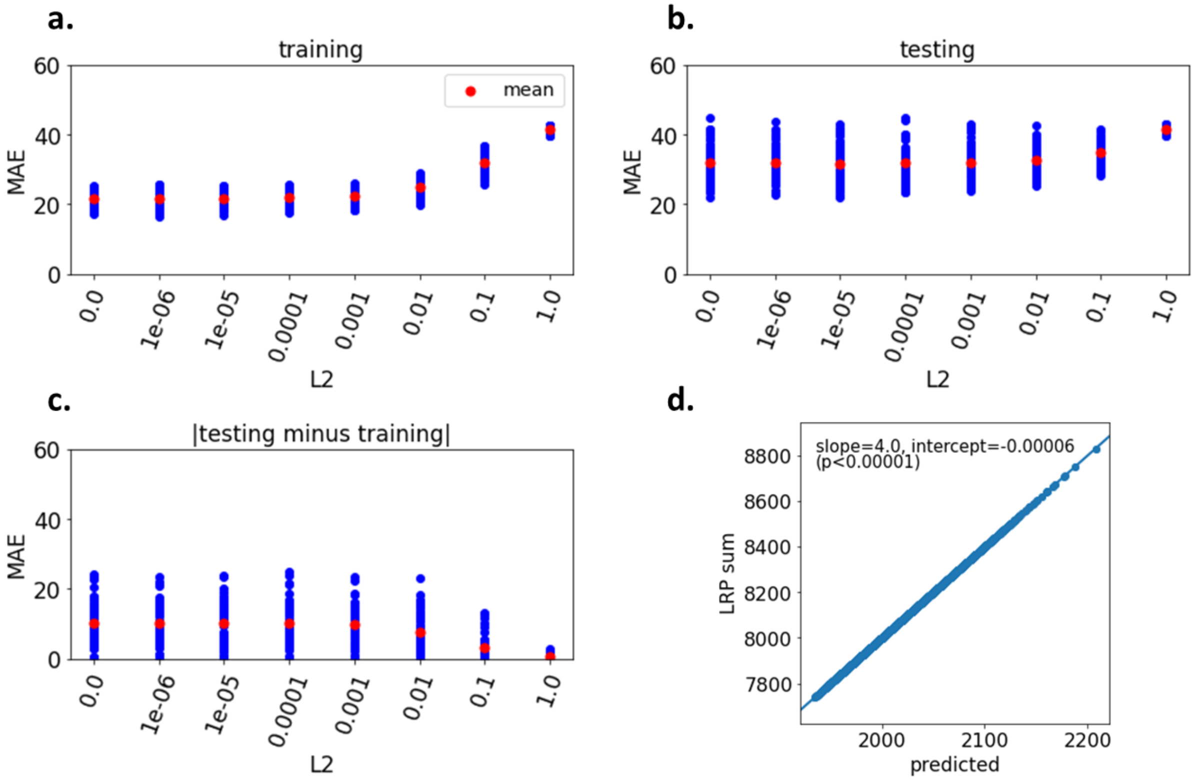

Supplementary Fig. 4. Training and testing results of the ANN.

(a-c) Mean Absolute Error (MAE) for different L2 regularization values from 51 different ANNs with different training/testing sets. MAE for training data (a), testing data (b) and absolute difference of testing minus training (c). (d) Predicted year vs sum of the relevance heatmap grid cell values obtained from $LRP_{\alpha2\beta1}$ for all models for a single ANN.

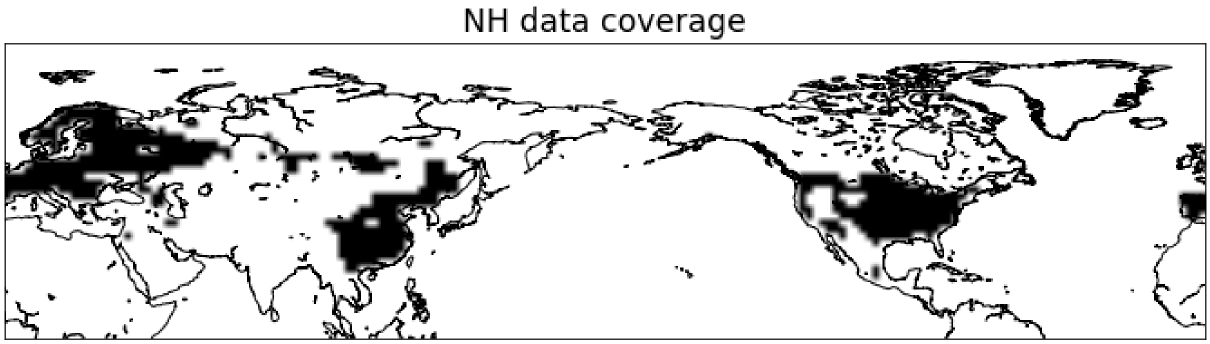

Supplementary Fig. 5. Northern hemisphere HadEX3 data coverage considered in the analysis. Only grids with a continuous data record for the period 1979-2018 (black) were selected and regridded to the common 2° x 2° spatial grid prior to the analysis.

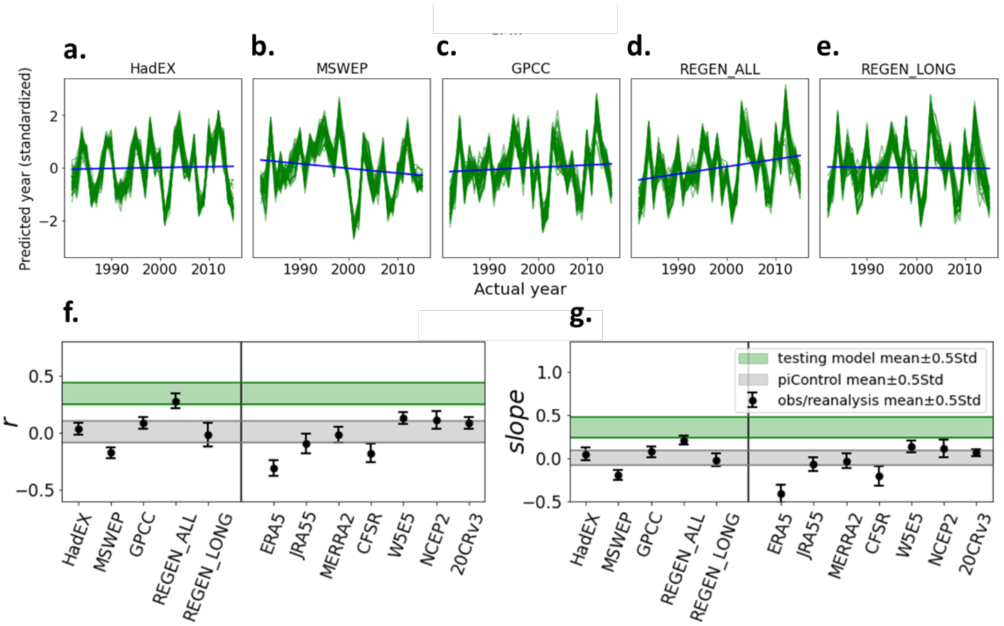

Supplementary Fig. 6. Same as Figure 3 but for the domain in Figure S5 and with HadEX3 based results.

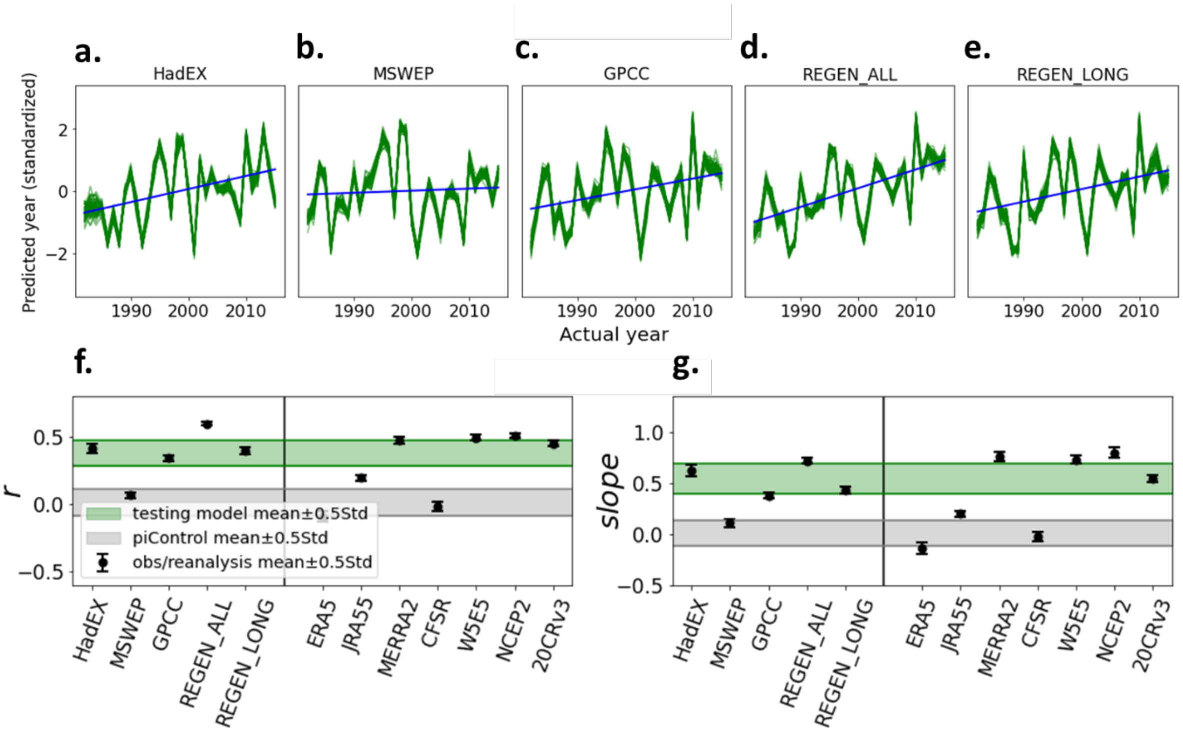

Supplementary Fig. 7. Same as Figure S6 but using CESM large ensemble to train the ANN.

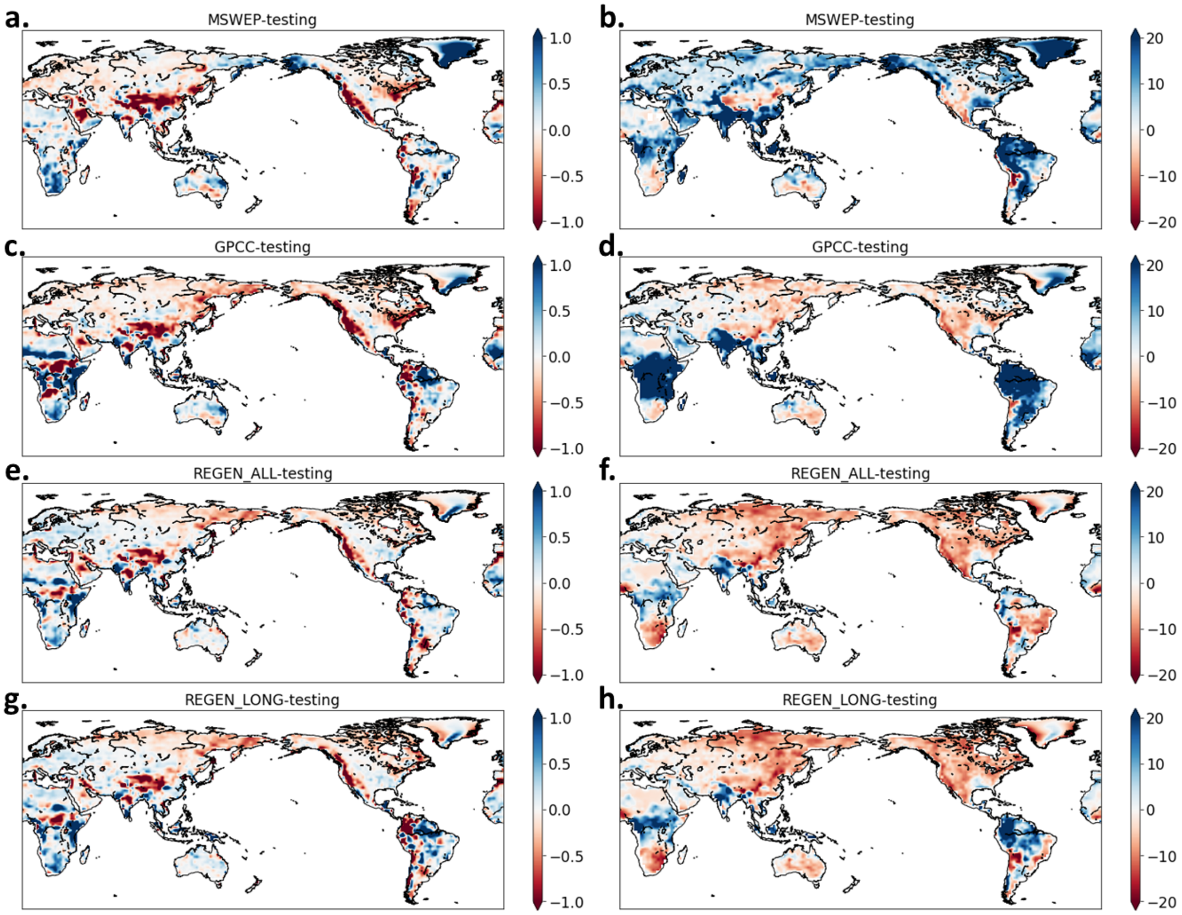

Supplementary Fig. 8. Differences of Rx1day and relevance between observations and models.

(a,c,e,g) Difference of time average relevance (unit: years) between observations and testing data. (b,d,f,h) Difference of time average Rx1day (unit: mm/day) between observations and testing data. For testing data, an average from all testing models of 51 different ANNs with different training/testing sets was obtained.

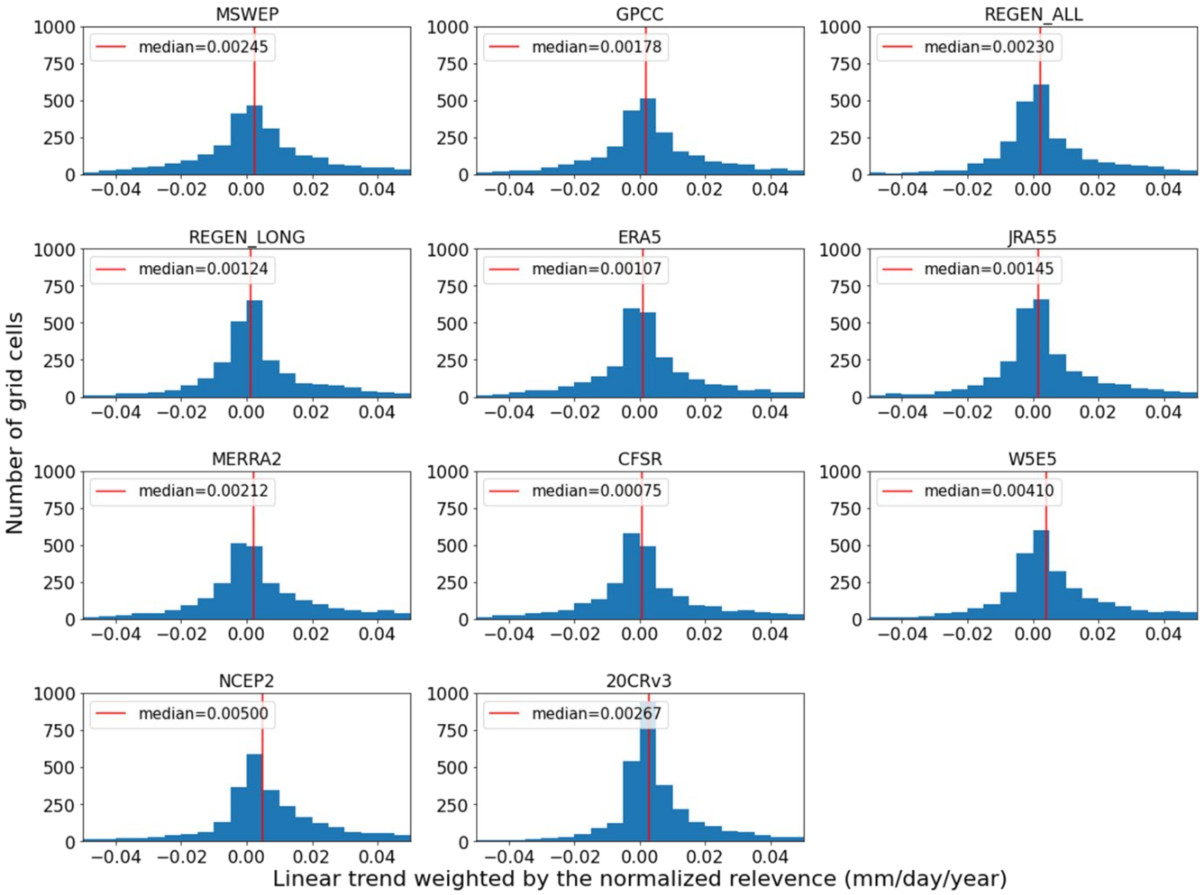

Supplementary Fig. 9. Histograms of the relevance-weighted linear trends (mm/day/year) in observed Rx1day for grid cells with positive relevance in Figure 1c. Bin width is 0.005 mm/day/year. Relevance value of each grid cell is first normalized by the global maximum and then used to weight the trend. Median of the distribution is depicted in a red vertical line. As shown by the median, datasets with a smaller anthropogenic signal (e.g. CFSR, ERA5, REGEN_LONG, as shown in Figure 3) have a lower number of grid cells with an increasing relevance-weighted trend in Rx1day compared to the other datasets.

References

1. Toms, B. A., Barnes, E. A., & Ebert-Uphoff, I. (2020). Physically interpretable neural networks for the geosciences: applications to earth system variability. *Journal of Advances in Modeling Earth Systems*, **12**, e2019MS002002. https://doi.org/10.1029/2019MS002002
2. Bach, S., Binder, A., Montavon, G., Klauschen, F., Müller, K.-R., & Samek, W. (2015). On pixel-wise explanations for non-linear classifier decisions by layer-wise relevance propagation. *Plos One*, **10**(7), e0130140. https://doi.org/10.1371/journal.pone.0130140
3. Montavon, G., Samek, W., & Müller, K. R. (2018). Methods for interpreting and understanding deep neural networks. *Digital Signal Processing*, **73**, 1– 15.
4. Montavon, G., Binder, A., Lapuschkin, S., Samek, W., & Müller, K. R. (2019). Layer-wise relevance propagation: an overview. In Explainable AI: interpreting, explaining and visualizing deep learning (pp. 193-209). Springer, Cham. doi: 10.1007/978-3-030-28954-6_10.
5. Hanczar, B., Zehraoui, F., Issa, T., & Arles, M. (2020). Biological interpretation of deep neural network for phenotype prediction based on gene expression. *BMC bioinformatics*, **21**(1), 1-18.
6. Dunn, R. J., Alexander, L. V., Donat, M. G., Zhang, X., Bador, M., Herold, N., ... & Brunet, M. (2020). Development of an updated global land in situ-based data set of temperature and precipitation extremes: HadEX3. *Journal of Geophysical Research: Atmospheres*, **125**(16), e2019JD032263.
7. Min, S. K., X. B. Zhang, F. W. Zwiers, and G. C. Hegerl (2011), Human contribution to more-intense precipitation extremes, *Nature*, **470**, 378– 381, doi:10.1038/nature09763
8. Paik, S., Min, S. K., Zhang, X., Donat, M. G., King, A. D., and Sun, Q. (2020). Determining the anthropogenic greenhouse gas contribution to the observed intensification of extreme precipitation. Geophysical Research Letters, **47**(12), e2019GL086875.
9. Kay, J. E., Deser, C., Phillips, A., Mai, A., Hannay, C., Strand, G., ... & Vertenstein, M. (2015). The Community Earth System Model (CESM) large ensemble project: A community resource for studying

climate change in the presence of internal climate variability. *Bulletin of the American Meteorological Society*, **96**(8), 1333-1349.

10. Shiu, C. J., Liu, S. C., Fu, C., Dai, A., and Sun, Y. (2012). How much do precipitation extremes change in a warming climate?. *Geophysical Research Letters*, **39**(17).
11. Sen, P. K. (1968), Estimates of the regression coefficient based on Kendall's tau. *Journal of the American statistical association*, **63**, 1379–1389.
12. Theil, H. (1950), A rank-invariant method of linear and polynomial regression analysis, *Indagationes Mathematicae*, **12**, 85–91.
13. Hamed, K. H., and A. R. Rao (1998), A modified Mann-Kendall trend test for autocorrelated data, *Journal of Hydrology*, **204**, 182– 196.
